# Nursing the Sick

**Published:** 1855-01

**Authors:** 


### NURSING THE SICK.

Who has not been sick? who has not needed nursing, or been called to give it to others? And what a blessing is good nursing in a world where disease abounds? There is reason to suppose that many die for the want of skillful attentions. The late Dr. Rush said that to be a good nurse was to be half of a physician. Few posts of domestic or private usefulness exceed that of a faithful nurse. In this light it should be habitually regarded.

The following suggestions are drawn from considerable experience. It is hoped they may not be without profit to some, especially the young and unskillful. They are not made in a vain and presumptuous spirit, but are prompted by the kindest feelings. Ignorance in a nurse cannot aid the recovery of the sick.

The first qualification of a good nurse is mental composure. In a disease there often occur sudden and violent changes, which require the calmest attention. If the invalid discovers trepidation in attendants, the effect will probably be injurious.

Presence of mind is always desirable, and often indispensable. The nurse is the helmsman of the craft that is now in dangerous waters, and must not yield to alarm. Much depends on the judgment of the nurse, whose mind must be calm, or disaster must follow. Too much solicitude is the bane of good nursing.

Firmness, united with gentleness, adds much to the influence and success of the nurse. The sick have many whims and caprices. To yield to all of these would be injurious. Yet in many things their wishes may be gratified. Never forget the debility and nervous distress induced by disease, loss of sleep, and want of exercise in the open air. Under no circumstances speak a harsh word to the sick. Be firm, but be gentle even to coaxing. Many a one is made worse by a cross word or look, when a smile or some sign of love would sooner have caused submission, and not have left an unhappy impression. Other words, besides those of heretics, do sometimes eat as doth a canker. They may make the sick wilful.

In a reverse, patience is essential. The natural effects of constant seclusion and anxious vigils are nervousness and irritability. But let not the sick suffer from such things. If you cannot command yourself, go and walk, or sleep, or do something to recreate, until you are able with pleasantness to resume your wearisome duties. This requires great forgetfulness of one's self. Be ingenious in relieving the monotony as well as the pains of the sufferer. Sickness renders one changeable and fastidious. Let us grant all reasonable wishes. None but those who have never endured the tedium of a long sickness, can hesitate to do every harmless thing which may possibly conduce to even the fancied or momentary relief of pain or despondency. Sometimes the nurse has a magisterial air, and is reluctant to yield to the little desires and ever-changing wants of an invalid. Patience and all long-suffering are required. An obliging disposition will be the result, and will produce the happiest effects.

Be always cheerful. Let your countenance betray no symptoms of gloom or depression. Strive to throw around you a bright and happy atmosphere. This itself will relieve much uncomfortable feeling in the sufferer.

Learn also to move quietly and speak gently in the sick-room. Tread lightly and let your voice be always modulated to a low but distinct tone. Even the rustling of paper, or the creak of a shoe or of a door-hinge, or the slightest noise sometimes produces great restlessness in the sick. Let every thing like noise be excluded, as far as possible.

Nothing adds more to the comfort of all confined to the sick-room than cleanliness. Put out of sight all medicines, and everything that can awaken unpleasant associations. Let everything be in its proper place. By always replacing necessary articles, the greatest neatness may be preserved without inconvenience to the sick or to the attendant. There should be no confusion. A quiet effort to keep the room in a neat and orderly condition will seldom injure the most feeble or the most excitable.

Let the nurse never lose vigilance. If it can be no longer preserved, let a temporary substitute be provided. Ever be on the alert. If possible, anticipate the wants of the sick. Try to save them the trouble of asking for what they wish. To invalids, unsolicited attentions are very grateful. Often they are so listless as to care for nothing. If possible, surprise them at times by something palatable and wholesome for nourishment.

Beware of annoying by your attentions. Civilities may be excessive and oppressive. In that case they are cruel to the sick. A well-balanced judgment, with some practice, will teach you when and how your services are required.

Some skill in cooking is, of course, a useful attainment in a nurse. The sick are often willing to take nourishment by the hand of love, while they would have refused the same from a stranger. This is specially true when savory food is unexpectedly presented.

To many these suggestions may seem useless, for they are conscious of having neither taste nor talent for nursing. True, one endowed by nature with so excellent a gift, is able to act with less constraint and more success than others. But experience and a real desire to learn will secure very wonderful results. Cultivate a love for the sick-room. At least, labor to be cheerful when duty calls you there.

By putting in practice these rules, together with the lessons of wisdom you may receive from older and wiser persons, you will find yourself at last able to discharge this otherwise sad and painful, but important duty with pleasure to yourself and comfort to those you love.

The judicious will easily supply many things omitted in the details of these rules.

BENIGNA.

